# Respiratory Sinus Arrhythmia Acts as a Moderator of the Relationship Between Parental Marital Conflict and Adolescents’ Internalizing Problems

**DOI:** 10.3389/fnins.2019.00500

**Published:** 2019-05-24

**Authors:** Sumaira Khurshid, Yuan Peng, Zhenhong Wang

**Affiliations:** ^1^School of Psychology, Shaanxi Normal University, Xi’an, China; ^2^Shaanxi Province Key Research Centre of Child Mental and Behavioral Health, Xi’an, China

**Keywords:** marital conflict, internalizing problems, respiratory sinus arrhythmia, adolescent, interaction

## Abstract

The present study examined the potential moderating role respiratory sinus arrhythmia (RSA) plays in the relationship between parental marital conflict and adolescents’ internalizing problems. To examine this issue, data were collected from 330 adolescents (13–14 years, 182 boys). The Chinese version of the Achenbach Youth Self-Report-2001 and the Chinese version of the Children’s Perception of Interparental Conflict were used to assess the adolescents’ internalizing problems and their perceptions of parental marital conflict. To obtain RSA data, electrocardiogram monitoring was performed on the adolescents at baseline and during a series of stress tasks (watching a film clip depicting marital conflict, a mental arithmetic task, and a speech task). The results indicated that baseline RSA and RSA reactivity to the film clip moderated the relationship between parental marital conflict and internalizing problems in early adolescents. The moderating effect of baseline RSA supported the BSCT hypothesis. Specifically, adolescents with low baseline RSA have both the highest and lowest levels of internalizing problems, depending on the level of marital conflict. In contrast, adolescents with high levels of baseline RSA have moderate levels in internalizing problems, regardless of the level of marital conflict they experience. Similarly, high marital conflict was related to internalizing problems for adolescents with less RSA suppression or RSA augmentation but not for those with greater RSA suppression. This effect was specific to stress related to marital conflict, as RSA reactivity to the mental arithmetic task and speech task did not moderate the relationship between marital conflict and internalizing problems. These findings suggest that certain profile of parasympathetic nervous activity is a risk factor for internalizing problems particularly for those who experience high-conflict environments.

## Introduction

Internalizing problems (e.g., anxiety, depression) are common in adolescents and predict pervasive impairment in relation to social adaptation and academic achievement (e.g., Forns et al., 2012; Scalco et al., 2014). Thus, from a prevention viewpoint, it is critical to investigate and identify related vulnerabilities and protective factors regarding such problems. The developmental psychopathology framework suggests that multiple environmental and individual risk factors contribute to the development of internalizing problems (Cummings and Davies, 2002; Saxbe et al., 2012; Barroso et al., 2018) and, among such environmental risk factors, marital conflict has begun to be regarded as an important adverse family factor related to internalizing problems in adolescents (Cummings and Davies, 2010; El-Sheikh et al., 2013). Similarly, parasympathetic nervous system (PNS) function, indexed by respiratory sinus arrhythmia (RSA) activity, has been regarded as a physiological protective or risk factor that decreases or increases internalizing problems (Dietrich et al., 2007; Porges, 2007; Gentzler et al., 2009; El-Sheikh et al., 2013). Several studies have explored RSA activity, including baseline RSA and RSA reactivity interact with marital conflict, through the Person × Environment perspective (Cicchetti, 2006), mainly in attempts to predict internalizing problems among children and adolescents (Katz and Gottman, 1997; Whitson and El-Sheikh, 2003; El-Sheikh and Whitson, 2006); however, the findings have been inconclusive. As a result, the present study was conducted to examine how baseline RSA and RSA reactivity to stress interact with marital conflict to predict adolescents’ internalizing problems. In particular, this study sought to clarify whether high or low baseline RSA is an indicator of high physiological reactivity to marital-conflict environments, and whether the interacting role of marital conflict and RSA reactivity in predicting adolescents’ internalizing problems is influenced by RSA reactivity measured during different tasks.

Marital conflict is defined as any difference, disagreement, or argument regarding an issue of family life, and this includes all kinds of psychological and physical conflicts (Cummings and Davies, 2002). Marital conflict is widely regarded as a core indicator of family solidarity and the key element in determining the quality of family life (Erel and Burman, 1995; Cummings and Davies, 2002). The emotional security theory (Davies and Cummings, 1994; Davies et al., 2002) proposes that marital conflict disrupts children’s and adolescents’ emotional security and increases their negative emotional and behavioral responses, thereby increasing their psychological maladjustment, including their risk of developing internalizing problems (Tu et al., 2016). Moreover, a large number of studies have demonstrated that marital conflict is associated with a broad array of adjustment problems in adolescents, such as academic difficulties, externalizing problems, and internalizing problems (for reviews, see Grych and Fincham, 2001; Davies and Lindsay, 2004; Cummings and Davies, 2010; Tu et al., 2016).

According to recent conceptual and empirical work, individual factors, such as certain profiles of PNS activity, may play a role in influencing the vulnerability of psychopathology including internalizing problems (Beauchaine, 2001; Porges, 2007). PNS activity can be assessed using a cardiac measure of RSA, which reflects rhythmic fluctuations in heart rate in relation to phases of the respiratory cycle (e.g., Porges, 1995; Gentzler et al., 2009). Baseline RSA (i.e., RSA level when in a resting state) and RSA reactivity (i.e., estimated by RSA change from baseline to a challenging state) are two commonly used RSA indices. Research has proposed that baseline RSA reflects a person’s ability to maintain organism homeostasis, focus attention in normal situations, and conduct social engagement (Porges, 1995, 2007), while RSA reactivity reveals the extent to which a person can respond flexibly to internal stimuli and external environmental changes (Porges, 1995, 2007; Thayer and Lane, 2000). RSA reactivity can be quantified as RSA suppression (decreased RSA when performing tasks as compared to that at baseline) or RSA augmentation (increased RSA when performing tasks as compared to that at baseline). Specifically, during a challenging task, RSA suppression is generally considered an index of individuals’ ability to adapt flexibly to environmental demands, which in turn reflects the physiological processes that help the individuals address the challenge and self-regulate their emotions and/or behaviors (Beauchaine, 2001; Calkins and Keane, 2004; Gentzler et al., 2009). RSA augmentation represents a heightened parasympathetic response, which is associated with hypervigilance and predicts problem behaviors such as internalizing problems (Katz, 2007; Graziano and Derefinko, 2013).

Many studies have found that lower baseline RSA is linked to internalizing problems (Forbes et al., 2006; Dietrich et al., 2007; Wei et al., 2017), while in contrast, other studies have found that lower baseline RSA is not linked to internalizing problems in children and adolescents (El-Sheikh et al., 2011; Hinnant and El-Sheikh, 2013). One possible explanation for these conflicting findings is that the association between baseline RSA and internalizing problems might partly be affected by environmental variables. Similarly, mixed findings have been found in regard to the association between RSA reactivity and internalizing problems. Some studies have found that greater RSA suppression is associated with less internalizing problems (Pearson et al., 2005; Gentzler et al., 2009), some other studies have observed contradictory results (Boyce et al., 2001; Hinnant and El-Sheikh, 2009), and another set of studies has found no direct link between RSA reactivity and internalizing problems such as depression in non-clinical samples (Hinnant and El-Sheikh, 2013; Koenig et al., 2016). One potential explanation for such mixed findings is that the association between RSA reactivity and adjustment outcomes largely depends on the characteristics of the environmental challenge in question (Porges, 2007; Obradović et al., 2011; Overbeek et al., 2014; Cui et al., 2015).

The diathesis-stress model and the biological sensitivity to context theory (BSCT; Boyce and Ellis, 2005) propose that individual temperament, genetics, or autonomic nervous system responses interact with the environment to exacerbate or attenuate an individual’s maladaptation (Belsky and Pluess, 2009; Obradoviæ et al., 2010). Considering that baseline RSA is associated with self-regulatory capacity, and that RSA reactivity during a task is associated with self-regulatory effort (Segerstrom and Nes, 2007; Thayer et al., 2009; Balzarotti et al., 2017), RSA activity has recently been considered a moderator between environment and an individual’s adaptation (e.g., El-Sheikh et al., 2001; Obradović et al., 2011). In contrast to the diathesis-stress model, which conceptualizes high reactivity as a vulnerability factor for maladjustment (Monroe and Simons, 1991), the BSCT proposes that high physiological stress reactivity reflects high biological sensitivity to context (Boyce and Ellis, 2005). It also posits that children with high physiological reactivity are more sensitive to both negative and positive environments. In other words, in an adverse environment, high physiological reactivity might intensify the risk of maladjustment, whereas in supportive and nurturing environments, it might result in positive adjustment. In contrast, low biological stress reactivity is less affected by the environment (Boyce and Ellis, 2005).

In this regard, whether high or low baseline RSA represents high physiological reactivity still remains an open question. Some researchers have suggested that high baseline RSA reflects greater physiological reactivity, as it facilitates flexible responses to stress, and the ability to adapt to environmental challenges (Thayer and Lane, 2000; Porges, 2007). However, some other researchers have proposed that low baseline RSA reflects high physiological reactivity (Eisenberg et al., 2012), as it is related to higher negative emotional reactivity and motor/affective reactivity, which have been viewed as indicators of susceptibility to the environment (Fabes and Eisenberg, 1997; Beauchaine, 2001; Kagan and Fox, 2007; Eisenberg et al., 2012). Therefore, whether high or low baseline RSA represents high physiological reactivity in response to marital-conflict environment is needed to be clarified further.

Several studies have identified the moderating effect of RSA reactivity on the relationship between marital conflict and internalizing problems (Katz and Gottman, 1997; El-Sheikh et al., 2001; Whitson and El-Sheikh, 2003; El-Sheikh and Whitson, 2006). In contrast to the BSCT, these studies have found that children and adolescents who showed greater RSA suppression while watching a mock adult argument were less affected by the negative effects of adverse family environments, whereas children and adolescents who showed less RSA suppression or RSA augmentation while watching the mock adult argument were more vulnerable to adverse family environments (Katz and Gottman, 1997; El-Sheikh et al., 2001; Whitson and El-Sheikh, 2003; El-Sheikh and Whitson, 2006). However, other studies have failed to find a moderating effect of RSA reactivity in the connection between marital conflict and internalizing problems, using both cognitive tasks and watching peer-bullying film clips as stressors (Obradović et al., 2011). This indicates, and previous studies have suggested, that the moderating effect of RSA reactivity on the relationship between family factors (including marital conflict) and individuals’ adjustment may largely depend on the characteristics of the laboratory challenge tasks used to elicit RSA reactivity (Obradović et al., 2011; Overbeek et al., 2014; Cui et al., 2015). Therefore, it is important to examine whether the characteristics of the stress tasks used to evoke RSA reactivity affect the relationship between family environment and psychological adaptation in children and adolescents. Considering this, in the present study, watching a film clip depicting marital conflict, a mental arithmetic task, and a speech task were used to explore interaction effects between RSA reactivity and marital conflict in regard to predicting internalizing problems. These tasks were chosen because they are common stress-induction stimuli used in reactivity protocols (Dickerson and Kemeny, 2004; Lü et al., 2017).

The present study was performed on a sample of early adolescents. Early adolescence has been regarded as a critical period during which individuals’ biological and psychological states undergo marked developmental changes and they face a series of challenges, such as more complicated school tasks (Cicchetti and Rogosch, 2002). Previous studies have suggested that there is also a marked increase in internalizing problems during this crucial developmental stage (Angold et al., 2002); therefore, the present study focused on internalizing problems in early adolescents.

In addition, there is mixed evidence for gender difference in the moderating effect of RSA on the relationship between marital conflict and internalizing problems (El-Sheikh and Erath, 2011). Therefore, the present study also examined whether the moderating effect of RSA activity on the relationship between parental marital conflict and adolescents’ internalizing problems differed by sex.

Overall, the present study was conducted to examine whether high or low baseline RSA represents high biological susceptibility to marital-conflict environment. This study also examined whether greater RSA suppression might function as a protective factor and moderate the association between marital conflict and adolescent’s internalizing problems, and whether the moderating effect of RSA suppression on the relationship between marital conflict and adolescents’ internalizing problems might depend on RSA reactivity measured during different challenge tasks.

## Materials and Methods

### Participants

Three hundred and forty-six junior high school students aged 13–14 years, all from two-parent families, were recruited from a city in northwest China. Their parents were married and were the students’ biological parents. Almost all participants (97%) were of Chinese Han ethnicity, and all were Mandarin Chinese speaking students. Of these, the data of six participants were excluded from the analysis because they did not complete the questionnaires, and the physiological data of 10 participants were not usable as a result of acquisition issues (e.g., equipment malfunction or electrode misplacement). Therefore, data from 330 participants [182 boys, mean age = 13.7 years, standard deviation (*SD*) = 0.8] were valid. They completed questionnaires that assessed internalizing problems and parental marital conflict, and then participated in a laboratory-based physiological experiment. All of the participants reported no history of cardiovascular disease and that they were not taking any medications that could interfere with the research results. All of the participants reported normal or corrected-to-normal vision.

The participants were asked to report their parents’ monthly income using a 4-point scale (1 = less than ¥3000, 2 = ¥3000-¥7000, 3 = ¥7000-¥10000, and 4 = more than ¥10000) and their parents’ education level using a 7-point scale (1 = lower than elementary school, 2 = elementary school, 3 = junior high school, 4 = high school, 5 = college or university, 6 = master’s degree, 7 = doctoral degree). The participants’ socioeconomic status (SES) was obtained by summing the standard scores (*M* = 0, *SD* = 1) of the following three variables according to previous studies (Schulting et al., 2005; Cohen et al., 2006): (a) the father’s education level (*M* = 2.64, *SD* = 0.92); (b) the mother’s education level (*M* = 2.60, *SD* = 0.94); and (c) household income (*M* = 2.88, *SD* = 0.39). Among the participants’ parents, 59% of mothers and 71% of fathers had an educational attainment ranging from vocational school to a college or university degree. Most of their parents worked outside the home in occupations ranging from blue collar to professional. The mean monthly combined family income was between about ¥7000 and ¥10000. The sample was well representative of school adolescents in urban China. All participants were compensated ¥60 (approximately $10) for their participation.

### Procedure

This study was approved by the Institutional Review Board of the Psychology School of Shaanxi Normal University. Written informed consent was obtained from the parents of all participants prior to data collection, and the participants were informed of the nature of the study and were told that there was no penalty for not participating. The experiment was conducted from 2:00 pm to 5:00 pm every day for 4 weeks, with each participant attending for a single day. The participants were invited into a brightly lit, quiet room, which was equipped with computers. Before the formal test, detailed instructions were provided to ensure that the participants clearly understood the experimental procedures. All participants were instructed to refrain from performing physical exercise or consuming any caffeine or alcohol for 2 h before the commencement of the experiment, which was in order to eliminate the risk of any exogenous effects on the physiological measures. After completing the questionnaires, electrocardiogram (ECG) recording electrodes (SOMNOtouch^TM^ device) were attached to the participants. The participants were then given 10 min to acclimate to the laboratory and to relax. After this, the formal physiological experiment began, with all instructions for the experimental procedure being simultaneously presented on a monitor screen.

**FIGURE 1:**
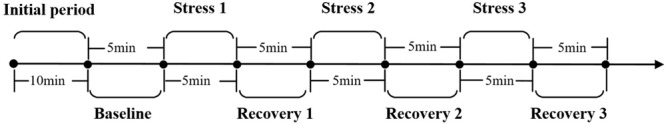
Time-line of experimental session.

The entire experiment included seven phases (see Figure 1), and after each phase, the participants were asked to rate their subjective emotional experience (SEE), including nervousness and anxiety, on two 5-point scales from 0 (“relaxed”) to 4 (“nervous” or “anxious”). First, the participants were shown a neutral picture on the computer screen (a picture of a cup, selected from the International Affective Picture System, IAPS; Lang et al., 2005), which was designed to keep them relaxing while ECG and respiration signals were recorded; this enabled the measurement of the participants’ 5-min baseline RSA values. Second, the participants were instructed to watch a film clip depicting marital conflict, which served to induce a stress response. Third, the participants were given 5 min for rest (recovery period 1), during which they were instructed to sit, relax, and view neutral pictures (e.g., a picture of a cup or an umbrella also selected from the IAPS; Lang et al., 2005). Fourth, the participants were requested to complete a mental arithmetic task (Diamond et al., 2012). Fifth, the participants rested again (recovery period 2). Sixth, the participants were instructed to give a speech. Seventh, the participants rested again (recovery period 3).

In order to reduce the impact of order effects, the sequences of the three stress tasks were balanced across the entire experiment. Specifically, one third of the participants watched the marital-conflict film clip first, then performed the mental arithmetic task second, and performed the speech task third; one third performed the mental arithmetic task first, the speech task second, and watched the marital-conflict film clip third; and the other third performed the speech task first, watched the marital-conflict film clip second, and performed the mental arithmetic task third.

### Stress Tasks

#### Watching a Marital-Conflict Film Clip Task

The participants were instructed to watch a 5-min film clip featuring marital conflict. The suitability of the conflict film, featuring verbal and physical conflict between a couple, was previously assessed through a preliminary experiment. Pre-experimental testing showed that the watching the film clip task induced higher subjective and physiological responses^1^.

#### Mental Arithmetic Task

The participants were informed that their mathematical skills would be evaluated based on their performance in the task, in which they were asked to subtract 13 from a series of four-digit numbers as fast and accurately as possible. Every 4.5 s, the correct number would be displayed on the monitor, accompanied by a beep sound. The participants were requested to state the result of their calculation before the correct answer was displayed on the monitor after the beep. For avoiding social-evaluative threat, the two research assistants who were present with them left the experiment room when the participants performed the mental arithmetic task.

#### Speech Task

The participants were told to give a speech for a mock class-leader election. Two research assistants served as live interviewers for each speech. The participants were provided with the following instruction (originally given in Chinese): “You will deliver a speech for a class-leader election, for which you have 120 s to prepare; then, you will have 5 min to state the type of position you are running for, as well as the reasons you qualify for this position. Your performance will be evaluated by the research assistants in terms of overall content, clarity, and delivery.” After the 120-s preparation phase, the participants delivered their 5-min speech to the two assistants. During the speech period, whenever the participants paused for more than 10 s, they were prompted to continue. The validity of the social stress task has been verified in a previous study (Lü et al., 2016).

### Measures

#### Marital Conflict

The conflict characteristics subscale of the Chinese version of the Children’s Perception of Interparental Conflict (the original instrument was developed by Grych and Fincham, 1990, and the Chinese version was revised by Chi and Xin, 2003) was used to measure parental marital conflict. This subscale includes three dimensions: conflict frequency (six items; e.g., “I often see my parents arguing”), conflict intensity (seven items; e.g., “when my parents have an argument, they yell a lot”), and conflict resolution (six items; e.g., “even after my parents stop arguing, they stay mad at each other”). The three dimensions are summed to create a single overall measure of parental marital conflict, with higher scores indicating greater parental marital conflict. Responses are given using a 5-point scale ranging from 1 (“never”) to 5 (“always”). For the present study, the Cronbach’s α for this subscale was 0.86.

#### Internalizing Problems

Internalizing problems were assessed using the Chinese Version of the Achenbach Youth Self-Report-2001 (Achenbach and Rescorla, 2001; Wang et al., 2016). The internalizing problem subscale contains 30 items that measure withdrawal/depressed, anxious/depressed, and somatic complaints, which are then summed to create a single overall measure of internalizing problems; higher scores indicate more internalizing problems. Sample items include “I worry a lot” and “I am unhappy, sad, or depressed.” Responses are provided using a 3-point scale ranging from 0 (“not true”) to 2 (“very true or often true”). For the present study, the Cronbach’s α for the internalizing subscale was 0.88.

#### Physiological Data

The ECG data were continuously recorded using SOMNOtouch^TM^ RESP (SOMNOmedics, Germany), with a sampling rate of 256 Hz. Disposable Ag-AgCl electrodes were attached to each participant’s lower left rib and right and left clavicle. The ECG sensors were subsequently connected to the SOMNOtouch^TM^ RESP.

The ECG data were then transferred into DOMINO light software 1.4.0 (SOMNOmedics, Germany) for automatic detection of artifacts (which were to be discarded from the analysis). In data preparation, the R-R intervals were resampled at 4 Hz and detrended based on the smoothness prior approach (Tarvainen et al., 2002). RSA was quantified via a high-frequency interbeat-interval power spectrum (0.22–0.40 Hz for adolescents aged 13 years and 0.20–0.40 Hz for adolescents aged 14; Shader et al., 2018) corresponding to the respiratory cycle, and the values were natural log transformed (ln) to fit the assumption of linear analyses, yielding ln units (ms^2^). RSA during each study phase was averaged to compute mean level for each experimental period (i.e., baseline RSA and RSA reactivity). Additionally, a standardized residual score was computed to remove overlap between baseline RSA and RSA reactivity scores (Calkins and Keane, 2004). RSA reactivity was computed as the standardized residual of the RSA value that was predicted for each stress task based on the RSA value during the baseline period (Hastings et al., 2008; Burt and Obradoviæ, 2013). A positive standardized residual score would indicate a significant increase from baseline (RSA augmentation), while a negative standardized residual score would indicate a significant decrease from baseline (RSA suppression; Hastings et al., 2008).

### Analytic Strategy

Outliers, +/-3 *SD* from the mean, were identified for the study variables. One adolescent had very low baseline RSA, three had a very strong RSA suppression response to speech task, and two had a very strong RSA augmentation response to the speech task. All of the outlier data points were replaced with the next value present in the data (Wilcox, 2012), and the main findings were not changed by using this approach.

First, to test whether the challenging tasks successfully induced participants’ stress reactivity, within-subject comparisons of subjective nervousness, subjective anxiety and RSA data obtained during baseline, the watching the film clip task, the mental arithmetic task, the speech task, and the recovery periods were performed with separate repeated measures analyses of variance (ANOVAs). Pairwise comparisons were conducted using Bonferroni correction. Greenhouse-Geisser corrections would be applied if the assumption of sphericity was violated.

All statistical tests were conducted using SPSS 22 (IBM, United States). First, difference scores (the absolute change between baseline and RSA response to each challenge) analysis was conducted to report the general variation trends in RSA from baseline to the stress task periods. Second, Pearson correlations were computed to examine the associations among the study variables. Third, separate multiple regression analyses were used to examine the main effects of marital conflict and RSA variables (baseline RSA and RSA reactivity) and the interaction effects on adolescents’ internalizing problems; all the predictor variables were mean-centered prior to conducting regression analyses (Aiken and West, 1991). In each regression, the main effects of sex, marital conflict, and baseline RSA (or RSA reactivity) were entered in the first step, and the two-way interaction terms (i.e., marital conflict × baseline RSA) were entered in the second step. The three-way interaction of sex × marital conflict × baseline RSA (or RSA suppression) was entered in the third step. A power analysis indicated that to detect a three-way interaction with a medium effect size (i.e., 0.15), a sample size of at least 89 is needed. Thus, our sample size is comparably large and can be used to reliably detect a three-way interaction. To evaluate the significant interaction, the procedures outlined by Aiken and West (1991) were used to plot the predicted outcome variable for levels of the independent variable (ranging from -1 *SD* to +1 *SD*) at both high and low levels of the moderator (ranging from -1 *SD* to +1 *SD*).

## Results

### Descriptive Statistics

The means and standard deviations of the variables are provided in Table 1. Compared to boys (*M* = 10.33, *SD* = 7.70), girls had greater internalizing problems (*M* = 14.50, *SD* = 9.14). No other difference was observed between boys and girls.

**Table 1:** Descriptive statistics among variables.

	Mean	*SD*
Marital conflict	13.39	3.64
Baseline RSA	6.32	1.15
RSAR^a^ (mental arithmetic)	–0.002	0.58
RSAR^a^ (speech)	–0.008	0.61
RSAR^a^ (film clip)	–0.01	0.66
Internalizing problems	12.13	8.56

*RSAR = RSA reactivity. Sex was coded 0 for males and 1 for females. ^*a*^Standardized residual score (with baseline RSA partialed out), ^∗^*p* < 0.05, ^∗∗^*p* < 0.01, ^∗∗∗^*p* < 0.001.*

During the watching the film clip task, 156 (47%) children displayed RSA augmentation, and 174 (53%) displayed RSA suppression. During the mental arithmetic task, 128 (39%) children displayed RSA augmentation, and 202 (61%) displayed RSA suppression. During the speech task, 125 (38%) children displayed RSA augmentation, and 205 (62%) displayed RSA suppression.

### Analyses of Variance (ANOVAs) by Task

Regarding the watching the marital-conflict film clip task, the repeated measures ANOVAs showed significant main effects for subjective nervousness [*F*(1.99, 640.92) = 74.34, *p* < 0.001, *η^2^_p_* = 0.19], subjective anxiety [*F*(1.98, 638.48) = 45.75, *p* < 0.001, *η^2^_p_* = 0.12], and RSA [*F*(1.79, 585.64) = 23.31, *p* < 0.001, *η^2^_p_* = 0.10]. *Post hoc* tests indicated that the levels of subjective nervousness and anxiety were greater during the challenge task than during the baseline period [*t*(329) = 7.96, *p* < 0.001; *t*(329) = 8.51, *p* < 0.001] and the recovery period [*t*(329) = 10.18, *p* < 0.001; *t*(329) = 8.00, *p* < 0.001]. Similarly, *post hoc* tests also showed that RSA decreased significantly from baseline to the challenge task period [*t*(329) = 5.62, *p* < 0.001], and then increased significantly during the recovery period [*t*(329) = 3.72, *p* < 0.01]. These results showed that using the watching the marital-conflict film clip task was effective in inducing changes in individuals’ subjective and physiological reactivity.

Regarding the mental arithmetic task, the repeated measures ANOVAs significant main effects for subjective nervousness [*F*(1.96, 640.14) = 166.04, *p* < 0.001, *η^2^_p_* = 0.34], subjective anxiety [*F*(1.98, 642.22) = 161.96, *p* < 0.001, *η^2^_p_* = 0.55], and RSA [*F*(1.92, 619.04) = 49.71, *p* < 0.001, *η^2^_p_* = 0.13]. *Post hoc* tests indicated that the levels of subjective nervousness and anxiety were greater during the challenge task than during the baseline period [*t*(329) = 11.03, *p* < 0.001; *t*(325) = 10.18, *p* < 0.001] and the recovery period [*t*(329) = 12.12, *p* < 0.001; *t*(329) = 13.08, *p* < 0.001]. Similarly, *post hoc* tests also showed that RSA decreased significantly from baseline to the challenge task period [*t*(329) = 4.11, *p* < 0.001], and then increased significantly during the recovery period [*t*(329) = 2.87, *p* < 0.01]. These results showed that using a mathematical challenge task was also effective in inducing changes in individuals’ subjective and physiological reactivity.

Finally, with regard to the speech task, the repeated measures ANOVA showed significant main effects for subjective nervousness [*F*(1.84, 157.98) = 179.13, *p* < 0.001, *η^2^_p_* = 0.36], subjective anxiety [*F*(1.94, 629.59) = 93.50, *p* < 0.001, *η^2^_p_* = 0.22], and RSA [*F*(1.74, 589.61) = 50.63, *p* < 0.001, *η^2^_p_* = 0.14]. *Post hoc* tests indicated that the levels of subjective nervousness and anxiety were greater during the challenge task than during the baseline period [*t*(329) = 9.80, *p* < 0.001; *t*(329) = 8.80, *p* < 0.001] and the recovery period [*t*(329) = 10.23, *p* < 0.001; *t*(329) = 9.21, *p* < 0.001]. Similarly, *post hoc* tests also showed that RSA decreased significantly from baseline to the challenge task period *[t*(329) = 4.24, *p* < 0.001], and then increased significantly during the recovery period *[t*(329) = 2.05, *p* < 0.05]. These findings showed that using a speech task was also effective in inducing changes in individuals’ subjective and physiological reactivity.

### Correlations Analyses

Correlations among study variables were presented in Table 2. As hypothesized, exposure to marital conflict was related to a high level of internalizing problems. Moreover, baseline RSA and RSA reactivity to the three challenge tasks were not related to internalizing problems as main effects.

**Table 2:** Correlation among variables.

	1	2	3	4	5	6	7	8
1 Sex	1							
2 SES	–0.04	1						
3 Marital conflict	0.09	–0.13*	1					
4 Baseline RSA	0.02	–0.08	0.02	1				
5 RSAR^a^ (mental arithmetic)	0.001	0.04	0.05	–0.06	1			
6 RSAR^a^ (speech)	0.10	0.05	0.02	–0.08	0.52**	1		
7 RSAR^a^ (film clip)	0.002	0.04	0.08	–0.12	0.50**	0.42**	1	
8 Internalizing problems	0.25**	0.03	0.19**	0.06	–0.06	0.03	–0.04	1

### Predictions for Internalizing Problems Based on Marital Conflict and Baseline RSA

**FIGURE 2:**
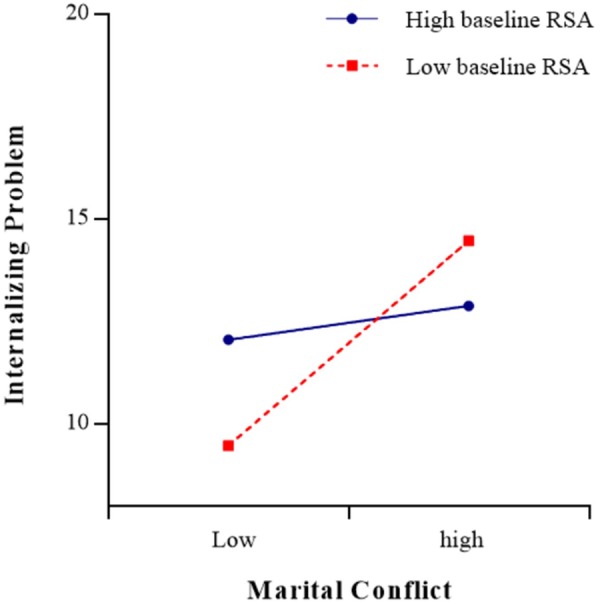
The association between marital conflict and adolescents’ internalizing problems at low (–1 *SD*) and high (+1 *SD*) levels of baseline RSA.

**FIGURE 3:**
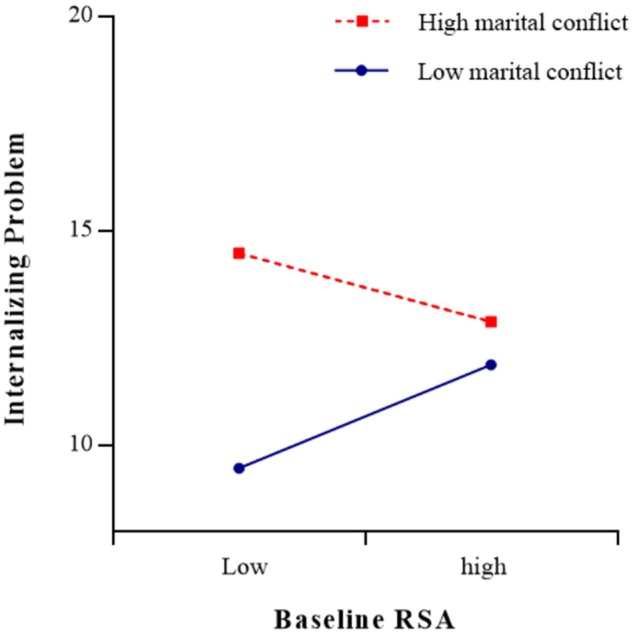
The association between baseline RSA and adolescents’ internalizing problems at low (–1 *SD*) and high (+1 *SD*) levels of marital conflict.

The results of the regression analyses were presented in Tables 3, 4. As shown in Table 3, the moderating effect of baseline RSA (*t* = -2.26, *p* < 0.05) on the relationship between marital conflict and internalizing problems was significant (see Figure 2, 3). The simple slope test found that for adolescents with low baseline RSA, marital conflict significantly predicted internalizing problems [simple slope = 2.50, *SE* = 0.06, *t*(329) = 0.62, *p* < 0.001], but for adolescents with high baseline RSA, marital conflict did not have significant effect on internalizing problems [simple slope = 0.62, *SE* = 0.05, *t*(329) = 3.85, *p* > 0.05]. The three-way interaction role of sex × marital conflict × baseline RSA was not significant (*p* > 0.05).

**Table 3:** Main effect and interactive effect of marital conflict and baseline RSA.

	Internalizing problems					
	B	*SE*	β	*t*	95% CI for *B*	Δ*R*^2^
**Step 1**						0.07
Marital conflict	1.30	0.48	0.15	2.71**	[0.36, 2.24]	
Baseline RSA	0.25	0.47	0.03	0.53	[-0.68, 1.18]	
Sex	1.91	0.46	0.22	4.17***	[1.01, 2.81]	
**Step 2**						0.09
Marital conflict × Baseline RSA	–1.20	0.47	–0.14	–2.56*	[-2.12, -0.28]	
Marital conflict × Sex	0.04	0.47	0.005	0.09	[-0.87, 0.95]	
Baseline RSA × Sex	0.83	0.43	0.10	1.75	[-0.10, 1.76]	
**Step 3**						0.09
Marital conflict × Baseline RSA × Sex	0.24	0.44	0.03	0.51	[-0.68, 1.15]	
	Total *R*^2^ = 0.11, *F*(7, 322) = 5.38^∗∗∗^					

**Table 4:** Main effect and interactive effect of marital conflict and RSAR (watching film clip).

	Internalizing problems					
	B	*SE*	β	*t*	95% CI for *B*	Δ*R*^2^
**Step 1**						0.08
Marital conflict	1.36	0.48	0.16	2.84**	[0.42, 2.30]	
RSAR	–0.43	0.46	–0.05	0.53	[-0.68, 1.18]	
Sex	2.05	0.46	0.24	4.48***	[1.15, 2.95]	
**Step 2**						0.11
Marital conflict ×RSAR	–1.05	0.48	–0.12	–2.17*	[-2.00, -0.10]	
Marital conflict × Sex	0.21	0.47	0.03	0.46	[-0.70, 1.13]	
RSAR × Sex	0.75	0.45	0.08	1.62	[-0.16, 1.67]	
**Step 3**						0.11
Marital conflict × RSAR × Sex	–0.71	0.47	–0.09	–1.52	[-1.64, 0.21]	
	Total *R*^2^ = 0.11, *F*(7, 322) = 5.86^∗∗∗^					

### Predictions for Internalizing Problems Based on Marital Conflict and RSA Reactivity

**FIGURE 4:**
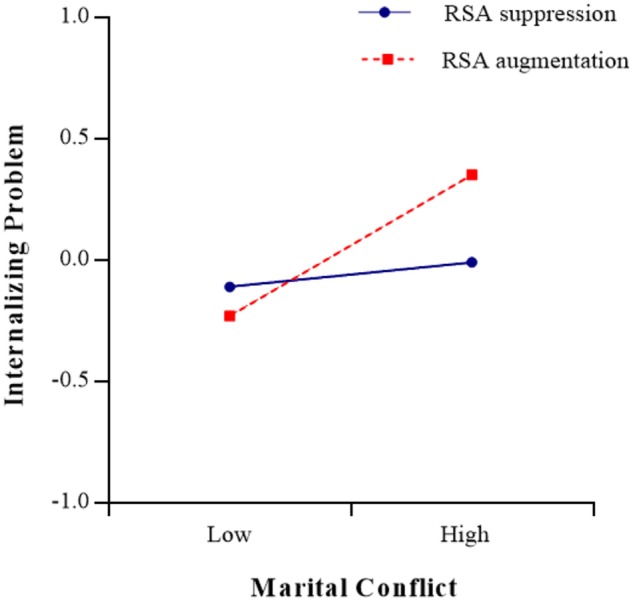
The association between marital conflict and adolescents’ internalizing problems at low (–1 *SD*) and high (+1 *SD*) levels of RSA reactivity under watching film clip task.

**FIGURE 5:**
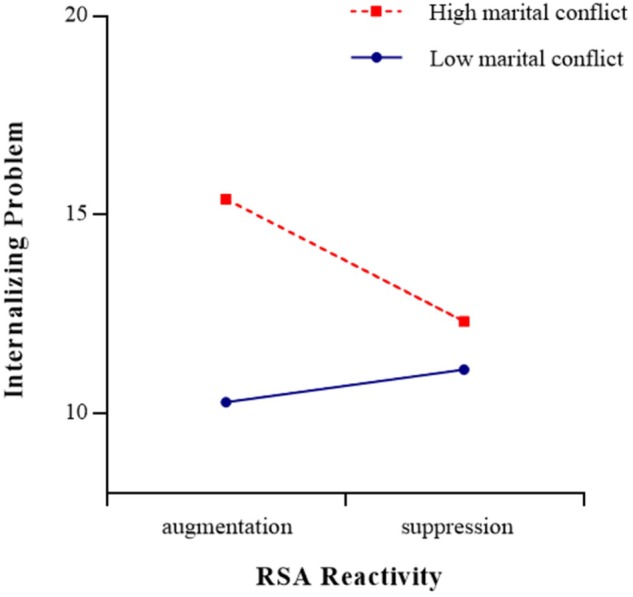
The association between RSA reactivity (watching film clip task) and adolescents’ internalizing problems at low (–1 *SD*) and high (+1 *SD*) levels of marital conflict.

As shown in Table 4, RSA reactivity to the marital-conflict film clip (*t* = -2.34, *p* < 0.05) had a significant moderating effect on the relationship between marital conflict and internalizing problems (see Figure 4, 5). The simple slope test found that for adolescents with RSA augmentation, marital conflict significantly predicted internalizing problems [simple slope = 2.55, *SE* = 0.06, *t*(329) = 3.76, *p* < 0.001], but for adolescents with greater RSA suppression, marital conflict did not have significant effect on internalizing problems [simple slope = 0.61, *SE* = 0.06, *t*(329) = 0.92, *p* > 0.05]. The three-way interaction role of sex × marital conflict × RSA reactivity (watching film clip) was not significant (*p* > 0.05). Meanwhile, as shown in Tables 5, 6, RSA reactivity to the mental arithmetic and speech tasks (*ps* > 0.05) did not have significant moderating effects on the relationship between marital conflict and internalizing problems. The three-way interaction roles of sex × marital conflict × RSA reactivity (mental arithmetic and speech tasks) were not significant (*ps* > 0.05).

**Table 5:** Main effect and interactive effect of marital conflict and RSAR (mental arithmetic task).

	Internalizing problems					
	B	*SE*	β	*t*	95% CI for *B*	Δ*R*^2^
**Step 1**						0.09
Marital conflict	1.46	0.47	0.17	3.12**	[0.54, 2.38]	
RSAR	–0.43	0.46	–0.05	0.53	[-0.68, 1.18]	
Sex	2.05	0.46	0.24	4.48***	[1.15, 2.95]	
**Step 2**						0.01
Marital conflict × RSAR	–0.76	0.46	–0.09	–1.64	[-1.67, 0.15]	
Marital conflict × Sex	–0.18	0.46	–0.02	–0.40	[-1.11, 0.73]	
RSAR × Sex	0.81	0.47	0.09	1.72	[-0.12, 1.75]	
**Step 3**						0.00
Marital conflict × RSAR × Sex	0.06	0.46	0.008	0.14	[-0.84, 0.97]	
	Total *R*^2^ = 0.11, *F*(7, 322) = 5.86^∗∗∗^		

**Table 6:** Main effect and interactive effect of marital conflict and RSAR (speech task).

	Internalizing problems					
	B	*SE*	β	*t*	95% CI for *B*	Δ*R*^2^
**Step 1**						0.09
Marital conflict	1.51	0.47	0.17	3.22**	[0.58, 2.44]	
RSAR	0.04	0.46	0.005	0.098	[-0.87, 0.96]	
Sex	1.94	0.46	0.22	4.22***	[1.03, 2.84]	
**Step 2**						0.008
Marital conflict ×RSAR	–0.16	0.46	–0.02	–0.34	[-1.06, 0.74]	
Marital conflict × Sex	–0.11	0.47	–0.01	–0.24	[-1.03, 0.81]	
RSAR × Sex	0.77	0.46	0.09	1.63	[-0.15, 1.69]	
**Step 3**						0.004
Marital conflict × RSAR × Sex	0.58	0.45	0.06	1.25	[-0.32, 1.47]	
	Total *R*^2^ = 0.10, *F*(7, 322) = 5.21^∗∗∗^					

## Discussion

The present study examined the associations among parental marital conflict, adolescents’ RSA variables (baseline RSA and RSA reactivity), and adolescents’ internalizing problems. The results showed that the adolescents’ baseline RSA and RSA reactivity in response to a film clip depicting marital conflict moderated the relationship between parental marital conflict and adolescents’ internalizing problems.

Consistent with previous studies (Cummings and Davies, 2010; El-Sheikh et al., 2013), the present study found that marital conflict is positively related to adolescents’ internalizing problems, that is, high marital conflict is related to more internalizing problems in adolescents. According to the emotional security theory (Davies and Cummings, 1994; Davies et al., 2002), marital conflict disrupts adolescents’ emotional security, causes negative emotional responses, undermines their psychological adjustment, and increases the likelihood of internalizing problems (Tu et al., 2016). The present study together with previous studies (Cummings and Davies, 2010; El-Sheikh et al., 2013) demonstrate that high parental marital conflict has a negative association with children’s psychological functioning and may elevate the risk of adolescents’ internalizing problems.

Similar to some previous studies (El-Sheikh and Erath, 2011; Hinnant and El-Sheikh, 2013; Koenig et al., 2016), the present study did not find any direct association between baseline RSA or RSA reactivity and adolescents’ internalizing problems. However, consistent with previous studies (Katz and Gottman, 1997; El-Sheikh and Erath, 2011), the present study found that baseline RSA has a significant moderating effect on the relationship between marital conflict and adolescents’ internalizing problems. The moderating effect of baseline RSA supported the BSCT hypothesis. The moderating effect indicated that adolescents with low baseline RSA have low levels of internalizing problems only if they lived in low-conflict environments. Low baseline RSA reflected a low threshold for autonomic nervous system arousal, which may facilitate adolescents’ sensitivity to the support and resources from positive family environments. However, in a high-conflict family, such adolescents were also biological vulnerable to the negative effect of their parents’ psychological and physical conflicts and had little protection from the risk of internalizing problems. In contrast, adolescents with high levels of baseline RSA have moderate levels in internalizing problems, regardless of the level of marital conflict they experience. Adolescents with high baseline RSA were more likely to maintain calm and to have the ability to adaptively regulate their emotions and behavior and were better able to cope with marital conflict, and may be at less risk of internalizing problems in high-conflict environments.

Moreover, consistent with previous studies (Whitson and El-Sheikh, 2003; El-Sheikh and Whitson, 2006), the present study found that RSA reactivity in response to a film clip depicting marital conflict moderated the relationship between marital conflict and adolescents’ internalizing problems and the moderating effect didn’t support the BSCT hypothesis. In other words, adolescents who showed appropriate RSA responses (greater RSA suppression) during marital conflict appear to be protected from internalizing problems, even when marital conflict was high, whereas adolescents who showed inappropriate RSA responses (either RSA augmentation or less RSA suppression) to marital conflict film clip were only protected from internalizing problems if they lived in a low-conflict environment (a more peaceful family). In the context of marital conflict, adolescents with greater RSA suppression might be able to better regulate their physiological arousal and emotional response and may be at less risk of internalizing problems (e.g., Whitson and El-Sheikh, 2003; El-Sheikh and Whitson, 2006). In contrast, adolescents with either RSA augmentation or less RSA suppression tended to be hypervigilant to negative emotional environments and might exhibit poor emotional regulation (Katz, 2007; Graziano and Derefinko, 2013), and such adolescents were more likely to develop internalizing problems in high-conflict environments.

In addition, considering that moderating effects are symmetrical, the results also suggested that marital conflict has significant moderating effects on the relationship between RSA activity (baseline RSA and RSA reactivity in response to marital-conflict film clip) and adolescents’ internalizing problems. The moderating effects indicated that in a high marital-conflict environment, adolescents with lower baseline RSA or RSA augmentation exhibited the most internalizing problems, and in a low marital-conflict environment, adolescents with lower baseline RSA exhibited the least internalizing problems; however, adolescents with higher baseline RSA or greater RSA suppression exhibited less internalizing problems even lived in a high marital-conflict environment. Therefore, in families with parental conflict, more attention should be paid to signs of internalizing problems in adolescents with more limited physical resources.

Despite the above, the present study did not find RSA reactivity to the mental arithmetic and speech tasks have significant moderating effects on the relationship between marital conflict and adolescents’ internalizing problems. These findings indicated that, as previous researchers have suggested, the moderating effects of RSA reactivity on the relationship between adverse family environments and adolescents’ maladaptive outcomes might vary with the characteristics of the laboratory stress tasks applied (Porges, 2007; Obradović et al., 2011; Overbeek et al., 2014; Cui et al., 2015). Previous studies have suggested that the mental arithmetic and speech tasks can be categorized as motivated performance tasks, while watching a film clip can be categorized as a passive task (Blascovich and Mendes, 2000), and that these stressors activate different cognitive and affective processes and have different central nervous system underpinnings (Dickerson and Kemeny, 2004). Moreover, it is plausible that the moderating effects depend on a match between RSA reactivity to the particular task and the task-related predictor. Watching a marital-conflict film clip closely resembles a parental marital-conflict situation that many adolescents witness in their daily lives, RSA reactivity to a marital-conflict film clip might be consistent with the RSA reactivity they usually experience during their parent’s conflict, and this might partly cause explain the present study observation that RSA reactivity to the marital-conflict film clip had a significant moderating effect on the relationship between marital conflict and adolescents’ internalizing problems.

The present study also examined whether the interactions between marital conflict and RSA activity in the prediction of adolescents’ internalizing problems differed by sex. Consistent with previous studies, the present study found that girls had greater internalizing problems than boys (Nolen-Hoeksema and Girgus, 1994), while no other difference was noted between boys and girls. These results suggested that higher baseline RSA and greater RSA suppression might protect both boys and girls against internalizing problems associated with marital conflict, and low baseline RSA among boys and girls might reflect their biological susceptibility to both negative and positive family environments.

Several limitations to the present study should be considered. First, although the findings indicated that baseline RSA and RSA reactivity interact with marital conflict to predict internalizing problems, as a result of the cross-sectional nature of the present study, no conclusion regarding causality or the direction of the effect can be drawn. Thus, future longitudinal research must explore the temporal relations among these variables. Second, marital conflict scores were obtained through adolescents’ self-reports. Although previous studies have noted that children’s and adolescents’ self-reports of marital conflict are more consistent predictors of adjustment than are parents’ reports of marital conflict (Grych et al., 2000), future research should include parents’ reports of marital conflict to fully evaluate the effect of family environments on adolescents. Third, the present study did not measure interpersonal violence at home. Future research should measure this factor as it is known to make adolescents feel less safe at home and lead to the development of more internalizing problems. Fourth, a low level of marital conflict does not necessarily indicate a supportive and nurturing environment; further studies should explore the issues that exist in positive family environments. Finally, the present study examined the moderating roles of RSA activity on the relationship between marital conflict and early adolescents’ internalizing problems. Future researchers should extend the findings to samples with a broader age range such as elementary and high school students to improve the generalizability of the results.

## Conclusion

In conclusion, the present study identified the moderating effects of PNS activity (baseline RSA and RSA reactivity) on the relationship between parental marital conflict and internalizing problems in early adolescents. It provided evidence indicating that poor parasympathetic regulation (low baseline RSA and inappropriate augmentation to a stressor) is a risk factor for internalizing problems particularly for those who experience high-conflict environments. It also provided preliminary evidence suggesting that the moderating effect of baseline RSA supported the BSCT hypothesis. Moreover, it indicated that the moderating effect of RSA reactivity on the relationship between parental marital conflict and early adolescents’ internalizing problems partly depend on the RSA reactivity measured during different challenge tasks.

## Ethics Statement

The authors assert that all procedures contributing to this work comply with the ethical standards of the relevant national and institutional committees on human experimentation and with the Helsinki Declaration of 1975, as revised in 2008. This study was approved by the Institutional Review Board of the Psychology School of Shaanxi Normal University.

## Author Contributions

All authors listed have made a substantial, direct and intellectual contribution to this work, and approved it for publication.

## Conflict of Interest Statement

The authors declare that the research was conducted in the absence of any commercial or financial relationships that could be construed as a potential conflict of interest.

## Footnotes

1 Pre-experimental testing with a sample of 32 participants (boys = 16, mean age = 12.8 years) showed that the film clip depicting marital conflict elicited greater subjective nervousness [*t*(30) = 2.34, *p* = 0.031, *d* = 1.08], subjective anxiety [*t*(30) = 2.34, *p* = 0.031, *d* = 1.08], and RSA stress reactivity [*t*(30) = 2.23, *p* = 0.039, *d* = 1.04] compared to the scores before watching the film clip.

## References

[B1] Achenbach T. M. Rescorla L. A. (2001). *Manual for the ASEBA School-Age Forms and Profiles.* Burlington, VT: University of Vermont.

[B2] Aiken L. S. West S. G. (1991). *Multiple regression: Testing and interpreting interactions.* Thousand Oaks, CA: Sage Publications Inc.

[B3] Angold A. Erkanli A. Silberg J. Eaves L. Costello E. J. (2002). Depression scale scores in 8-17-year-olds: effects of age and gender. *J. Child Psychol. Psychiatry* 43 1052–1063. 10.1111/1469-7610.00232 12455926

[B4] Balzarotti S. Biassoni F. Colombo B. Ciceri M. R. (2017). Cardiac vagal control as a marker of emotion regulation in healthy adults: a review. *Biol. Psychol.* 130 54–66. 10.1016/j.biopsycho.2017.10.008 29079304

[B5] Barroso N. E. Mendez L. Graziano P. A. Bagner D. M. (2018). Parenting stress through the lens of different clinical groups: a systematic review and meta-analysis. *J. Abnorm. Child Psychol.* 46 449–461. 10.1007/s10802-017-0313-6 28555335 PMC5725271

[B6] Beauchaine T. (2001). Vagal tone, development, and gray’s motivational theory: toward an integrated model of autonomic nervous system functioning in psychopathology. *Dev. Psychopathol.* 13 183–214. 10.1017/S0954579401002012 11393643

[B7] Belsky J. Pluess M. (2009). Beyond diathesis-stress: differential susceptibility to environmental influences. *Psychol. Bull.* 135 885–908. 10.1037/a0017376 19883141

[B8] Blascovich J. Mendes W. B. (2000). “Challenge and threat appraisals: the role of affective cues,” in *Feeling and Thinking: the Role of Affect in Social Cognition* ed. Forgas J. (Cambridge: Cambridge University Press) 59–82.

[B9] Boyce W. T. Ellis B. J. (2005). Biological sensitivity to context: I. An evolutionary-developmental theory of the origins and functions of stress reactivity. *Dev. Psychopathol.* 17 271–301. 10.1017/S0954579405050145 16761546

[B10] Boyce W. T. Quas J. Alkon A. Smider N. A. Essex M. J. Kupfer D. J. (2001). Autonomic reactivity and psychopathology in middle childhood. *Brit. J. Psychiatry* 179 144–150. 10.1192/bjp.179.2.144 11483476

[B11] Burt K. B. Obradoviæ J. (2013). The construct of psychophysiological reactivity: statistical and psychometric issues. *Dev. Rev.* 33 29–57. 10.1016/j.dr.2012.10.002

[B12] Calkins S. D. Keane S. P. (2004). Cardiac vagal regulation across the preschool period: STABILITY, continuity, and implications for childhood adjustment. *Dev. Psychobiol.* 45 101–112. 10.1002/dev.20020 15505799

[B13] Chi L. P. Xin Z. Q. (2003). The revision of children’s perception of marital conflict scale. *Chin. Men. Health* 17 554–556.

[B14] Cicchetti D. (2006). “Development and Psychopathology,” in *Developmental Psychopathology* Vol. 1 Theory and methods 2nd Edn eds Cicchetti D. Cohen D. J. (New York, NY: Wiley) 1–23

[B15] Cicchetti D. Rogosch F. A. (2002). A developmental psychopathology perspective on adolescence. *J. Consult. Clin. Psychol.* 70 6–20. 10.1037/0022-006X.70.1.6 11860057

[B16] Cohen S. Doyle W. J. Baum A. (2006). Socioeconomic status is associated with stress hormones. *Psychosom. Med.* 68 414–420. 10.1097/01.psy.0000221236.37158.b9 16738073

[B17] Cui L. Morris A. S. Harrist A. W. Larzelere R. E. Criss M. M. Houltberg B. J. (2015). Adolescent RSA responses during an anger discussion task: relations to emotion regulation and adjustment. *Emotion* 15 360–372. 10.1037/emo0000040 25642723 PMC4437810

[B18] Cummings E. M. Davies P. T. (2002). Effects of marital conflict on children: recent advances and emerging themes in process-oriented research. *J. Child Psychol. Psychiatry* 43 31–63. 10.1111/1469-7610.00003 11848336

[B19] Cummings E. M. Davies P. T. (2010). *Marital Conflict and Children: an Emotional Security Perspective.* New York, NY: Guilford Press.

[B20] Davies P. T. Cummings E. M. (1994). Marital conflict and child adjustment: an emotional security hypothesis. *Psychol. Bull.* 116 387–411. 10.1037/0033-2909.116.3.387 7809306

[B21] Davies P. T. Harold G. T. Goeke-Morey M. C. Cummings E. M. Shelton K. Rasi J. A. (2002). Child emotional security and interparental conflict. *Monogr. Soc. Res. Child Dev.* 67 121–127. 10.1111/1540-5834.00205 12528424

[B22] Davies P. T. Lindsay L. L. (2004). Interparental conflict and adolescent adjustment: why does gender moderate early adolescent vulnerability? *J. Fam. Psycho.* 18 160–170. 10.1037/0893-3200.18.1.160 14992618

[B23] Diamond L. M. Fagundes C. P. Cribbet M. R. (2012). Individual differences in adolescents’ sympathetic and parasympathetic functioning moderate associations between family environment and psychosocial adjustment. *Dev. Psychol.* 48 918–931. 10.1037/a0026901 22268602

[B24] Dickerson S. S. Kemeny M. E. (2004). Acute stressors and cortisol responses: a theoretical integration and synthesis of laboratory research. *Psychol. Bull.* 130 355–391. 10.1037/0033-2909.130.3.355 15122924

[B25] Dietrich A. Riese H. Sondeijker F. E. Greaves-Lord K. Ormel J. Neeleman J. (2007). Externalizing and internalizing problems in relation to autonomic function: a population-based study in preadolescents. *J. Am. Acad. Child Adolesc. Psychiatry* 6 378–386. 10.1097/CHI.0b013e31802b91ea 17314724

[B26] Eisenberg N. Sulik M. J. Spinrad T. L. Edwards A. Eggum N. D. Liew J. (2012). Differential susceptibility and the early development of aggression: interactive effects of respiratory sinus arrhythmia and environmental quality. *Dev. Psycho.* 48 755–768. 10.1037/a0026518 22182294 PMC3341487

[B27] El-Sheikh M. Arsiwalla D. D. Hinnant J. B. Erath S. A. (2011). Children’s internalizing symptoms: the role of interactions between cortisol and respiratory sinus arrhythmia. *Physiol. Behav.* 103 225–232. 10.1016/j.physbeh.2011.02.004 21315098 PMC3062736

[B28] El-Sheikh M. Erath S. A. (2011). Family conflict, autonomic nervous system functioning, and child adaptation: state of the science and future directions. *Dev. Psychopathol.* 23 703–721. 10.1017/S0954579411000034 23786705 PMC3695441

[B29] El-Sheikh M. Harger J. Whitson S. (2001). Exposure to parental conflict and children’s adjustment and physical health: the moderating role of vagal tone. *Child Dev.* 72 1617–1636. 10.1111/1467-8624.00369 11768136

[B30] El-Sheikh M. Keiley M. Erath S. Dyer W. J. (2013). Marital conflict and growth in children’s internalizing symptoms: the role of autonomic nervous system activity. *Dev. Psychol.* 49 92–108. 10.1037/a0027703 22448986 PMC3912743

[B31] El-Sheikh M. Whitson S. A. (2006). Longitudinal relations between marital conflict and child adjustment: vagal regulation as a protective factor. *J. Fam. Psychol.* 20 30–39. 10.1037/0893-3200.20.1.30 16569087

[B32] Erel O. Burman B. (1995). Interrelatedness of marital relations and parent-child relations: a meta-analytic review. *Psychol. Bull.* 118 108–132. 10.1037//0033-2909.118.1.108 7644602

[B33] Fabes R. A. Eisenberg N. (1997). Regulatory control and adults’stress-related responses to daily life events. *J. Pers. Soc. Psycho.* 73 1107–1117. 10.1037/0022-3514.73.5.1107 9364764

[B34] Forbes E. E. Fox N. A. Cohn J. F. Galles S. F. Kovacs M. (2006). Children’s affect regulation during a disappointment: psychophysiological responses and relation to parent history of depression. *Biol. Psychol.* 71 264–277. 10.1016/j.biopsycho.2005.05.004 16115722

[B35] Forns M. Abad J. Kirchner T. (2012). “Internalizing problems,” in *Encyclopedia of Adolescence* eds Roger J. R. Levesque J. D. (Berlin: Springer) 1464–1469.

[B36] Gentzler A. L. Santucci A. K. Kovacs M. Fox N. A. (2009). Respiratory sinus arrhythmia reactivity predicts emotion regulation and depressive symptoms in at-risk and control children. *Biol. Psychol.* 82 156–163. 10.1016/j.biopsycho.2009.07.002 19596044 PMC2848485

[B37] Graziano P. Derefinko K. (2013). Cardiac vagal control and children’s adaptive functioning: a meta-analysis. *Biol. Psychol.* 94 22–37. 10.1016/j.biopsycho.2013.04.011 23648264 PMC4074920

[B38] Grych J. H. Fincham F. D. (1990). Marital conflict and children’s adjustment: a cognitive-contextual framework. *Psychol. Bull.* 108 267–290. 10.1037/0033-2909.108.2.267 2236384

[B39] Grych J. H. Fincham F. D. (2001). *Interparental Conflict and Child Development: Theory, Research, and Applications.* New York, NY: Cambridge University Press.

[B40] Grych J. H. Jouriles E. N. Swank P. R. McDonald R. Norwood W. D. (2000). Patterns of adjustment among children of battered women. *J. Consult. Clin. Psychol.* 68 84–94. 10.1037//0022-006x.68.1.84 10710843

[B41] Hastings P. D. Sullivan C. McShane K. E. Coplan R. J. Utendale W. T. Vyncke J. D. (2008). Parental socialization, vagal regulation, and preschoolers’ anxious difficulties: direct mothers and moderated fathers. *Child Dev.* 79 45–64. 10.1111/j.1467-8624.2007.01110.x 18269508

[B42] Hinnant J. B. El-Sheikh M. (2009). Children’s externalizing and internalizing symptoms over time: the role of individual differences in patterns of RSA responding. *J. Abnorm. Child Psychol.* 37 1049–1061. 10.1007/s10802-009-9341-1 19711181

[B43] Hinnant J. B. El-Sheikh M. (2013). Codevelopment of externalizing and internalizing symptoms in middle to late childhood: sex, baseline respiratory sinus arrhythmia, and respiratory sinus arrhythmia reactivity as predictors. *Dev. Psychopathol.* 25 419–436. 10.1017/S0954579412001150 23627954 PMC3874140

[B44] Kagan J. Fox N. A. (2007). “Biology, Culture, and Temperamental Biases,” in *Handbook of Child Psychology: Vol. 3: Social, Emotional, and Personality Development* eds Eisenberg N. Damon W. Lerner R. L. (New York, NY: Wiley) 167–225.

[B45] Katz L. F. (2007). Domestic violence and vagal reactivity to peer provocation. *Biol. Psychol.* 74 154–164. 10.1016/j.biopsycho.2005.10.010 17118516 PMC2577379

[B46] Katz L. F. Gottman J. M. (1997). Buffering children from marital conflict and dissolution. *J. Clin. Child Psychol.* 26 157–171. 10.1207/s15374424jccp2602_4 9169376

[B47] Koenig J. Kemp A. H. Beauchaine T. P. Thayer J. F. Kaess M. (2016). Depression and resting state heart rate variability in children and adolescents - a systematic review and meta-analysis. *Clin. Psychol. Rev.* 46 136–150. 10.1016/j.cpr.2016.04.013 27185312

[B48] Lang P. J. Bradley M. M. Cuthbert B. N. (2005). *International Affective Picture System (IAPS): Affective Rating of Pictures and Instruction Manual.* Gainesville, FL: University of Florida Center for Research in Psychophysiology.

[B49] Lü W. Wang Z. H. Hughes B. M. (2016). Openness and physiological responses to recurrent social stress. *Int. J. Psychophysiol.* 106 135–140. 10.1016/j.ijpsycho.2016.05.004 27181704

[B50] Lü W. Xing W. Hughes B. M. Wang Z. H. (2017). Extraversion and cardiovascular responses to recurrent social stress: effect of stress intensity. *Int. J. Psychophysiol.* 131 144–151. 10.1016/j.ijpsycho.2017.10.008 29111452

[B51] Monroe S. M. Simons A. D. (1991). Diathesis-stress theories in the context of life stress research: implication for the depressive disorders. *Psychol. Bull.* 110 406–425. 10.1037/0033-2909.110.3.406 1758917

[B52] Nolen-Hoeksema S. Girgus J. S. (1994). The emergence of gender differences in depression during adolescence. *Psychol. Bull.* 115 424–443. 10.1037/0033-2909.115.3.424 8016286

[B53] Obradović J. Bush N. R. Boyce W. T. (2011). The interactive effect of marital conflict and stress reactivity on externalizing and internalizing symptoms: the role of laboratory stressors. *Dev. Psychopathol.* 23 101–114. 10.1017/S0954579410000672 21262042

[B54] Obradoviæ J. Bush N. R. Stamperdahl J. Adler N. E. Boyce W. T. (2010). Biological sensitivity to context: the interactive effects of stress reactivity and family adversity on socio-emotional behavior and school readiness. *Child Dev.* 81 270–289. 10.1111/j.1467-8624.2009.01394.x 20331667 PMC2846098

[B55] Overbeek T. J. M. van Boxtel A. Westerink J. H. D. M. (2014). Respiratory sinus arrhythmia responses to cognitive tasks: effects of task factors and RSA indices. *Biol. Psychol.* 99 1–14. 10.1016/j.biopsycho.2014.02.006 24561100

[B56] Pearson S. R. Alkon A. Treadwell M. Wolff B. Quirolo K. Boyce W. T. (2005). Autonomic reactivity and clinical severity in children with sickle cell disease. *Clin. Auton. Res.* 15 400–407. 10.1007/s10286-005-0300-9 16362543

[B57] Porges S. W. (1995). Orienting in a defensive world: mammalian modifications of our evolutionary heritage. A polyvagal theory. *Psychophysiology* 32 301–318. 10.1111/j.1469-8986.1995.tb01213.x 7652107

[B58] Porges S. W. (2007). The polyvagal perspective. *Biol. Psychol.* 74 116–143. 10.1016/j.biopsycho.2006.06.009 17049418 PMC1868418

[B59] Saxbe D. E. Margolin G. Spies Shapiro L. A. Baucom B. R. (2012). Does dampened physiological reactivity protect youth in aggressive family environments? *Child Dev.* 83 821–830. 10.1111/j.1467-8624.2012.01752.x 22548351 PMC3342838

[B60] Scalco M. D. Colder C. R. Hawk L. W. Read J. P. Wieczorek W. F. Lengua L. J. (2014). Internalizing and externalizing problem behavior and early adolescent substance use: a test of a latent variable interaction and conditional indirect effects. *Psychol. Addict. Behav.* 28 828–840. 10.1037/a0035805 25134030 PMC4165783

[B61] Schulting A. B. Malone P. S. Dodge K. A. (2005). The effect of school-based kindergarten transition policies and practices on child academic outcomes. *Dev. Psychol.* 41 860–871. 10.1037/0012-1649.41.6.860 16351333 PMC2757260

[B62] Segerstrom S. C. Nes L. S. (2007). Heart rate variability reflects self-regulatory strength, effort, and fatigue. *Psycho. Sci.* 18 275–281. 10.1111/j.1467-9280.2007.01888.x 17444926

[B63] Shader T. M. Gatzkekopp L. M. Crowell S. E. Jamila R. M. Thayer J. F. Vasey M. W. (2018). Quantifying respiratory sinus arrhythmia: effects of misspecifying breathing frequencies across development. *Dev. Psychopathol.* 30 351–366. 10.1017/S0954579417000669 28554343

[B64] Tarvainen M. P. Ranta-aho P. O. Karjalainen P. A. (2002). An advanced detrending method with application to HRV analysis. *IEEE. Bio-med. Eng.* 49 172–175. 10.1109/10.979357 12066885

[B65] Thayer J. F. Hansen A. L. Saus-Rose E. Johnsen B. H. (2009). Heart rate variability, prefrontal neural function, and cognitive performance: the neurovisceral integration perspective on self-regulation, adaptation, and health. *Ann. Behav. Med.* 37 141–153. 10.1007/s12160-009-9101-z 19424767

[B66] Thayer J. F. Lane R. D. (2000). A model of neurovisceral integration in emotion regulation and dysregulation. *J. Affect. Disord.* 61 201–216. 10.1016/S0165-0327(00)00338-4 11163422

[B67] Tu K. M. Erath S. A. El-Sheikh M. (2016). Coping responses moderate prospective associations between marital conflict and youth adjustment. *J. Fam. Psychol.* 30 523–532. 10.1037/fam0000169 26571195 PMC4868795

[B68] Wang R. C. Wang M. C. Gao Y. D. Jiang Y. L. Zhang X. C. Yao S. Q. (2016). Reliability and validity of the Chinese version of achenbach youth self-report (2001 version). *Chin. J. Clin. Psychol* 21 977–980. 10.16128/j.cnki.1005-3611.2013.06.036

[B69] Wei Z. Fagan S. E. Yu G. (2017). Respiratory sinus arrhythmia activity predicts internalizing and externalizing behaviors in non-referred boys. *Front. Psychol.* 8:1496. 10.3389/fpsyg.2017.01496 28955262 PMC5600989

[B70] Whitson S. M. El-Sheikh M. (2003). Moderators of family conflict and children’s adjustment and health. *J. Emot. Abus.* 3 47–73. 10.1300/J135v03n01_03

[B71] Wilcox R. R. (2012). *Introduction to Robust Estimation and Hypothesis Testing* 3rd Edn. Cambridge, MA: Academic Press.

